# Effect of the WWOX gene on the regulation of the cell cycle and apoptosis in human ovarian cancer stem cells

**DOI:** 10.3892/mmr.2015.3640

**Published:** 2015-04-17

**Authors:** HONGCHAO YAN, JIANYE TONG, XIAOMAN LIN, QIUYU HAN, HONGXIANG HUANG

**Affiliations:** Department of Obstetrics and Gynecology, The Affiliated Hospital of Xuzhou Medical College, Xuzhou, Jiangsu 221002, P.R. China

**Keywords:** WWOX gene, transfection, ovarian cancer, stem cells

## Abstract

In order to examine new ideas for gene therapy in ovarian cancer, the specific mechanism underlying the effects of the WW domain containing oxidoreductase (WWOX) gene on cell cycle regulation and apoptosis in human ovarian cancer stem cells was investigated. Ovarian cancer stem cells were transfected with a eukaryotic expression vector carrying the WWOX gene *in vitro* (recombinant plasmid) and cells transfected with the empty plasmid (empty plasmid) or untransfected cells were used as controls. Stably transfected cells were screened and amplified in culture and the WWOX protein was detected by western blot analysis in the three groups of cells. Western blot analysis was performed to detect the expression of cell cycle regulatory proteins cyclin E, cyclin-dependent kinase (CDK) 2, cyclin D1, CDK4 and apoptosis-related protein Wnt-5α and c-Jun N-terminal kinase (JNK), while polymerase chain reaction (PCR) was used to detect alterations in the mRNA expression levels of caspase-3. The results demonstrated that the WWOX protein was stably expressed in cells of the recombinant plasmid group, but was not detected in cells of the empty plasmid group and the control group. Cell proliferation at each time point decreased significantly in the recombinant plasmid group compared with the empty plasmid group and the control group. Flow cytometric analysis demonstrated that the proportion of cells in the G0/G1 phase in the recombinant plasmid group was significantly higher than that of cells in the empty plasmid group and the control group. The rate of apoptosis in the recombinant plasmid group was significantly higher than that of cells in the empty plasmid group and the control group. Western blot analysis demonstrated that the expression levels of cyclin E, CDK2, cyclin D1 and CDK4 in the recombinant plasmid group were significantly lower than those in the empty plasmid group and the control group; however, the expression levels of Wnt-5α and JNK were significantly higher than those in the empty plasmid group and the control group. PCR results demonstrated that the mRNA expression level of caspase-3 in the recombinant plasmid group was significantly higher than that in the empty plasmid group and the control group. In conclusion, the present study demonstrated that the WWOX gene can be stably expressed in ovarian cancer stem cells and that it inhibits the proliferation of ovarian cancer stem cells. The WWOX gene can downregulate the expression levels of cell cycle proteins cyclin E-CDK2 and cyclin D1-CDK4, which affects the cell cycle of ovarian cancer stem cells. Furthermore, the WWOX gene can upregulate the mRNA expression levels of Wnt-5α, JNK and caspase-3, thus contributing to apoptosis of ovarian cancer stem cells. The present study demonstrated that the WWOX gene may be an important molecular target for the treatment of ovarian cancer in the future.

## Introduction

Ovarian cancer has the highest mortality rate worldwide among various types of gynecological malignancy and has become a serious threat to female health due to difficulty in early diagnosis and poor treatment (1). There are multiple types of ovarian cancer, however, epithelial ovarian cancer is the most common type and is also the most harmful (2). Several studies on gynecological cancer have aimed to understand the biological behavior and mechanism of ovarian cancer in more depth so that better treatments can be developed (3–5). In previous years, with the progress in the study of cancer stem cytology, the biological behavior of ovarian cancer has been recognized from a new perspective.

In 2000, Weissman first proposed the cancer stem cell theory (6), in which cancer is recognized as a disease of stem cells and cancer stem cells are a class of cells with self-renewal capacity, capable of unlimited proliferation and differentiation, and recognized as the principal cause of tumorigenesis, abnormal proliferation, metastasis and recurrence. During the study of tumor cells in ascites from patients with ovarian cancer, Bapat *et al* (7) found a number of sphere-forming cells capable of suspended growth. These sphere-forming cells have a strong cloning capability *in vitro*; they can self-renew and are capable of self-renewal and differentiation into tumors of the same nature when seeded into nude mice, thus confirming the presence of ovarian cancer stem cells (8). In addition to *in vivo* and *in vitro* experiments, our group applied paclitaxel to cells suspended in culture in serum-free medium containing epidermal growth factor (EGF), basic fibroblast growth factor (bFGF), Noggin and leukemia inhibitory factor (LIF) to successfully screen ovarian cancer stem cells, with characteristic expression of CDl33^+^ and CD117^+^, and identified their specific markers and biological characteristics (9). Our previous study laid a solid foundation for the present study.

The WW domain containing oxidoreductase (WWOX) gene was initially isolated and identified as a tumor suppressor gene in 2000 by Bednarek *et al* (10), spanning the entire autosomal fragile site FRAl6D and promoting tumor progression through functional loss or protein inactivation. Gourley *et al* (11) demonstrated that the mRNA expression level of WWOX is significantly decreased in ovarian cancer cells compared with normal ovarian tissue, indicating that the WWOX gene can inhibit the occurrence of ovarian cancer.

To further investigate the effect of the WWOX gene on the biological behavior of ovarian cancer stem cells, the present study transfected ovarian cancer stem cells with the WWOX gene. The present study aimed to determine the effect of WWOX on the biological behavior of ovarian cancer stem cells and to identify the underlying mechanism in order to provide a theoretical basis for ovarian cancer gene therapy.

## Materials and methods

### Materials

Ovarian cancer stem cells and the pcDNA3.1-WWOX eukaryotic expression vector were provided by and stored at the Affiliated Hospital of Xuzhou Medical College (Xuzhou, China). The empty pcDNA3.1 plasmid was provided by Professor Shuqun Hu at the Research Center for Molecular Biology, Xuzhou Medical College. A liposome Lipofectamine 2000 transfection kit and G418 were purchased from Invitrogen Life Technologies (Carlsbad, CA, USA). Anti-WWOX (rabbit-anti-human monoclonal; 1:1,000; cat. no. 15800667461), cyclin E (goat-anti-rabbit monoclonal; 1:10,000; cat. no. 13764022678), cyclin-dependent kinase (CDK)2 (goat-anti-rabbit monoclonal; 1:10,000; cat. no. MAB4310), Wnt-5α (goat-anti-rabbit monoclonal; 1:10,000; cat. no. MA1-12192), p-JNK (goat-anti-rabbit monoclonal; 1:10,000; cat. no. 254515), cyclin D1 (goat-anti-rabbit monoclonal; 1:10,000; cat. no. AM1125a) and CDK4 (goat-anti-rabbit monoclonal; 1:10,000; cat. no. AP1486c) primary and secondary antibodies were purchased from Chemicon (Billerica, MA, USA). Engreen Cell propidium iodide (PI), an Engreen cell cycle and an Engreen apoptosis detection kits were purchased from Nanjing KeyGen Biotech., Co., Ltd. (Nanjing, China). The present study was approved by the Ethics Committee of The Affiliated Hospital of Xuzhou Medical College.

### Cell culture

Human ovarian cancer stem cells were cultured in serum-free medium containing EGF, bFGF, Noggin and LIF at 37°C, 5% CO_2_ and saturated humidity in a closed thermostat incubator.

### Transfection

The eukaryotic expression vector carrying the WWOX gene was transfected into ovarian cancer stem cells *in vitro* (recombinant plasmid), according to the manufacturer’s instructions of the Lipofectamine 2000 transfection kit. Stably-transfected cells were screened and later expanded. Ovarian cancer stem cells transfected with the empty plasmid (empty plasmid) and untransfected cells (control group) were used as controls. Following transfection, cells were screened and passaged in selective medium containing neomycin.

### Western blot analysis

The three groups of cells while in exponential growth phase were harvested and lysed in 200 *μ*l lysis buffer in ice. Following determining the protein concentration using a bicinchoninic acid assay kit (Sigma-Aldrich, St Louis, MO, USA), proteins were electrophoresed in 10% sodium dodecyl sulfate sodium-polyacrylamide gel and electrophoretically transferred onto nitrocellulose membranes. To detect WWOX protein, the membranes were blocked with 5% skim milk for 60 min, incubated with 1:1,000 anti-WWOX antibody (rabbit anti-human) at 4°C overnight and reacted with horseradish peroxidase-labeled goat anti-rabbit secondary antibody (1:10,000) at room temperature for 2 h. Enhanced chemical fluorescent emission (LumiPico^®^ ECL reagent; Invitrogen Life Technologies) reagents were added and the membranes were exposed and developed with a highly sensitive X-ray film in a dark room. Other cell cycle and apoptotic proteins were detected similarly as described above.

### MTT assay

As described above, the three groups of cells were seeded in 96-well plates at a density of 1.5×10^4^ cells/well. After 1, 2, 3, 4 and 6 days of culturing, 20 *μ*l MTT working solution was added and the cells were cultured for an additional 4 h in a CO_2_ incubator at 37°C. Dimethyl sulfoxide was added to stop the reaction and each well was measured for the optical absorbance at 490 nm (A) on an enzyme-linked immunosorbent detector (SCJ204; Alpha Innotech Corp., San Diego, CA, USA). The data were used to draw a cell growth curve.

### Determination of the cell cycle and apoptosis by flow cytometry

The three groups of cells were harvested and digested in 0.25% trypsin during the exponential growth phase. For cell cycle analysis, cells were washed with phosphate-buffered saline, centrifuged at 5,000 × g for 2 min, fixed in 70% ethanol overnight, dispersed in 100 *μ*l phosphate salt buffer to produce a single cell suspension and analyzed by flow cytometry. For apoptosis analysis, cells were washed with phosphate-buffered saline, centrifuged at 5,000 × g for 2 min, adjusted to 1.5×10^5^ cells/ml, stained with 5 *μ*l annexin V-fluorescein isothiocyanate and 5 *μ*l PI for 10 min in the dark at room temperature and analyzed by flow cytometry.

### Detection of caspase-3 mRNA by reverse transcription quantitative polymerase chain reaction (PCR)

Total RNA was extracted from the three groups of cells during the exponential growth phase using TRIzol reagent. The reverse transcription reaction was performed according to the manufacturer’s instructions (RT-PCR kit; Chemicon, Temecula, CA, USA). The following primer sequences were used: Caspase-3, forward 5′-GGTGTTGATGATGACATGGCG-3′ and reverse 5′-GTACCCTCTGCAGCATGAGAGTAG-3′ (amplified product: 419 bp); β-actin, forward 5′-TGGAGAAATCTGGCACCAC-3′ and reverse 5′-GAGGCGTACAGGGATAGCAC-3′ (amplified product: 295 bp). β-actin was used as an internal control. The reaction conditions were 94°C for 45 sec, 55°C for 60 sec and 72°C for 60 sec for 30 cycles followed by extension at 72°C for 10 min. The PCR-amplified products were electrophoresed on a 2% agarose gel, observed in a UV detector (4100; Olympus, Tokyo, Japan), photographed and recorded. The content of each amplified product was analyzed by the imaging software of an automatic gel electrophoresis analyzer Chemi Imager 5500 (Alpha Innotech Corp., San Diego, CA, USA). The content ratio of caspase-3 mRNA/β-actin mRNA represents the relative mRNA expression level of caspase-3.

### Statistical analysis

The data are presented as the mean ± standard error of the mean and were analyzed using SPSS 13.0 statistical software (SPSS, Inc., Chicago, IL, USA). The differences between two sets of data were compared by Student’s t-test. Differences among multiple data were compared by one-way analysis of variance. P<0.05 was considered to indicate a statistically significant difference.

## Results

### Alterations in the protein expression of WWOX in WWOX-transfected ovarian cancer stem cells

**Figure 1:**
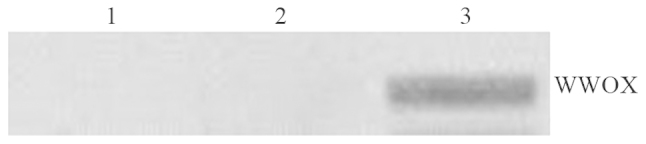
Detection of the WWOX protein in three groups of ovarian cancer stem cells by western blotting. 1, control group; 2, empty plasmid group; 3, recombinant plasmid group. WWOX, WW domain containing oxidoreductase.

Western blot analysis results demonstrated that the WWOX protein was highly expressed in the recombinant plasmid group, but was not detected in the empty plasmid group or the control group (Fig. 1).

### Alterations in cell proliferation in WWOX-transfected ovarian cancer stem cells

**Figure 2:**
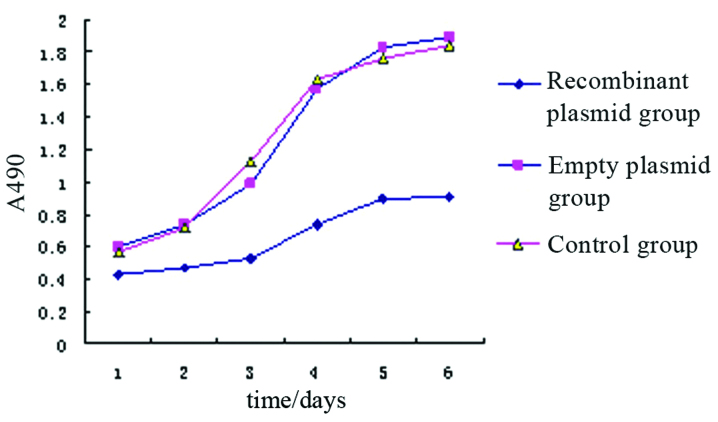
Growth curve of ovarian cancer stem cells following transfection of pcDNA3.1-WWOX.

The results of the MTT assay demonstrated that after 1, 2, 3, 4, 5 and 6 days of culturing, the number of cells in the recombinant plasmid group was 0.425±0.022, 0.471±0.032, 0.525±0.023, 0.741±0.023, 0.899±0.018 and 0.906±0.041, respectively, which was significantly reduced compared with those in the control group and the empty plasmid group at each time point (P<0.05). No statistically significant difference was identified between the control group and the empty plasmid group (P>0.05; Fig. 2).

### Alterations in the cell cycle in WWOX-transfected ovarian cancer stem cells

Flow cytometric analysis demonstrated that cells in the recombinant plasmid group were arrested at the G0/G1 phase and had a significantly reduced number of cells in the S phase compared with cells in the empty plasmid group and the control group. This difference was found to be statistically significant (P<0.05). However, no statistically significant difference was identified between the empty plasmid group and the control group (P>0.05; Table I).

### Alterations in apoptosis in WWOX-transfected ovarian cancer stem cells

**Figure 3:**
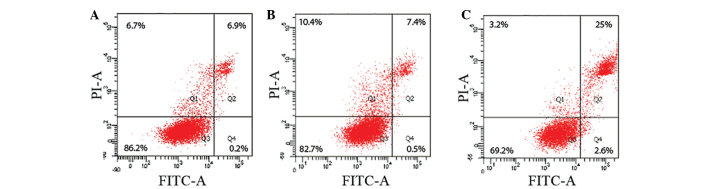
Apoptosis of three groups of cells following transfection of the WWOX gene. (A) Control group; (B) empty plasmid group; (C) recombinant plasmid. FITC, fluorescein isothiocyanate.

As shown by flow cytometry, the apoptotic rate in the recombinant plasmid group was 28.19%, which was significantly higher compared with that in the empty plasmid group (8.27%) and the control group (7.11%; P<0.05). No statistically significant difference was identified between the empty plasmid group and the control group (P>0.05; Fig. 3).

### Alterations in the expression of cell cycle regulatory proteins cyclin E, CDK2, cyclin D and CDK4 in WWOX-transfected ovarian cancer stem cells

**Figure 4:**
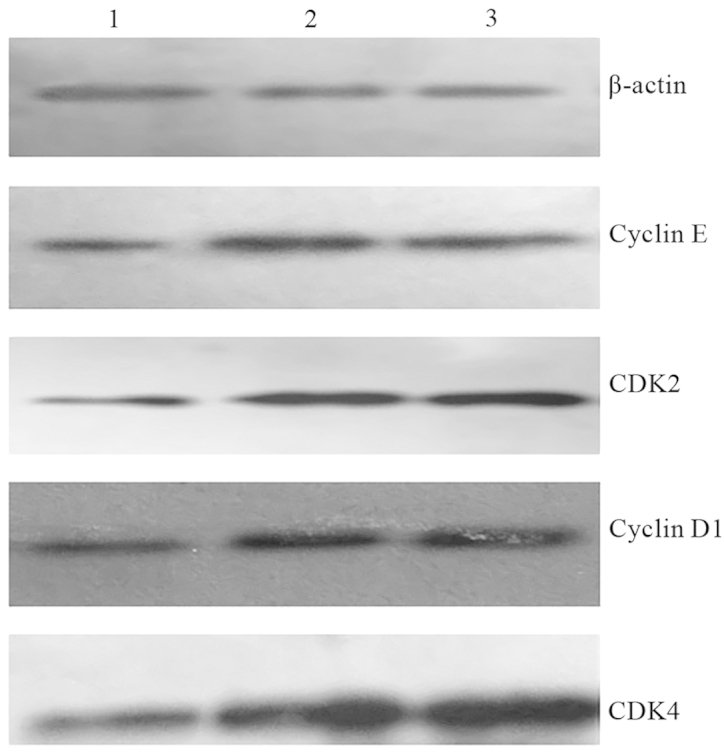
Detection of protein cyclin E, CDK2, cyclin D1 and CDK4 in three groups of cells by western blotting. 1, recombinant plasmid group; 2, empty plasmid group; 3, control group. CDK2, cyclin-dependent kinase 2.

The expression levels of cyclin E, CDK2, cyclin D1 and CDK4 in the recombinant plasmid group were: 1.033±0.012, 0.945±0.037, 1.068±0.057 and 1.296±0.016, respectively. These expression levels were significantly lower than that of the empty plasmid group (1.551±0.035, 2.547±0.026, 1.607±0.045, and 2.744±0.031, respectively) and the control group (1.546±0.015, 2.568±0.044, 1.601±0.028, and 2.795±0.012, respectively). These differences were statistically significant (P<0.05). However, no statistically significant difference was observed between the empty plasmid group and the control group (P>0.05; Fig. 4).

### Alterations in apoptosis-associated factors Wnt-5α and JNK and the mRNA expression of caspase-3 in WWOX-transfected ovarian cancer stem cells

**Figure 5:**
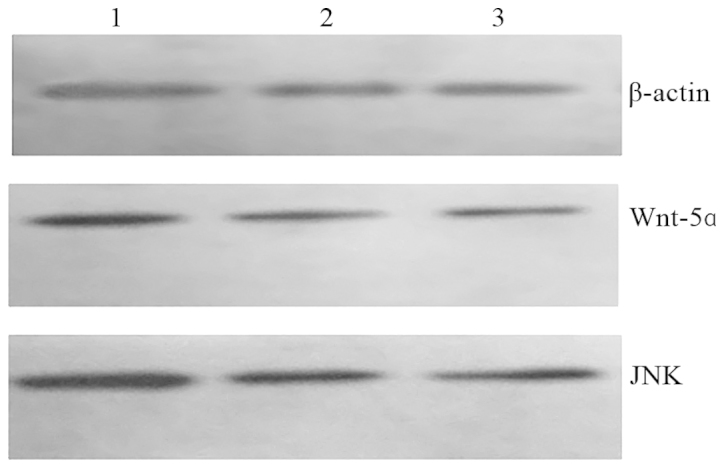
Detection of Wnt-5α and p-JNK protein in three groups of cells by western blotting. 1, recombinant plasmid group; 2, empty plasmid group; 3, control group. JNK, c-Jun N-terminal kinase.

Western blotting results demonstrated that the recombinant plasmid group had significantly higher protein levels of Wnt-5α and JNK (1.539±0.055 and 1.565±0.042, respectively) compared with the empty plasmid group (0.816±0.021 and 1.087±0.035, respectively) and the control group (0.849±0.007 and 1.085±0.026, respectively). The differences were statistically significant (P<0.05). No statistically significant difference was identified between the empty plasmid group and the control group (P>0.05; Fig. 5).

**Figure 6:**
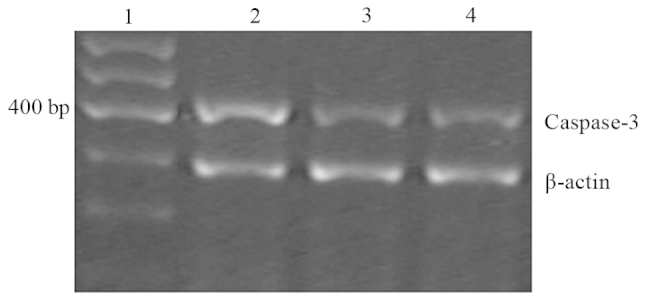
Detection of caspase-3 mRNA in three groups of cells by polymerase chain reaction. 1, marker; 2, recombinant plasmid group; 3, empty plasmid group; 4, control group.

The mRNA expression level of caspase-3 in the recombinant plasmid group was significantly higher than that in the empty plasmid group and the control group (P<0.05). While no statistically significant difference was identified between the empty plasmid group and the control group (P>0.05; Fig. 6).

## Discussion

Ovarian cancer is one of the most common types of female genital tumors worldwide. Due to the difficulty of early diagnosis in ovarian cancer, 60–70% of patients are diagnosed at an advanced phase (12). In addition, with poor treatment efficacy for advanced cases, ovarian cancer ranks first in gynecological cancer-associated mortality and is a severe threat to female health (13).

Cancer stem cells are a class of multipotent cells capable of self-renewal, unlimited proliferation and differentiation; however, they account for <1% of total tumor cells (14). During the cancer stem cell proliferation process and through uneven division, cancer stem cells divide to form a new tumor stem cell and another cell that may eventually differentiate into daughter cells of various types of cells, including tumor cells. The result of this process is the maintenance of a stable number of cancer stem cells and also the formation of a tumor (15). As increasing evidence supports the cancer stem cell theory (16–18), it has been hypothesized that ovarian cancer may also be a disease of stem cells. Zhang *et al* (19) successfully isolated ovarian cancer initiating cells from human serous ovarian cancer specimens and found that these cells can self-renew, form a suspension sphere and differentiate into tumors of the same nature when these sphere-forming cells were inoculated into mice *in vivo*, thus confirming the presence of ovarian cancer stem cells in ovarian cancer.

The WWOX protein contains two WW domains at the N-terminus (double tryptophan domain) that can easily bind proline-rich structures to participate in protein-protein interactions and one short-chain dehydrogenase/reductase domain at the C-terminus (oxidoreductase area) (20). Several studies have demonstrated that the WWOX gene is important in malignant tumors, particularly in the development of hormone-dependent tumors (21–23). One study demonstrated that normal ovarian tissue expressed high levels of the WWOX protein, however, the expression was reduced or absent in ovarian cancer patients; furthermore, a loss of WWOX expression was detected in 70% of ovarian mucinous adenocarcinoma and 42% of clear cell carcinoma (24). A previous study also found that epithelial ovarian carcinoma exhibited low expression levels of WWOX mRNA and in epithelial ovarian cancer and ovarian borderline tumor tissues, the positive expression rate and mRNA expression levels of WWOX were significantly lower than that in benign ovarian tumors and normal ovarian tissues (25). To further investigate the effect of the WWOX gene on the biological behavior of ovarian stem cells, the present study transfected ovarian cancer stem cells with WWOX by using the eukaryotic expression carrier pcDNA3.1-WWOX and detected cell proliferation by using an MTT assay in WWOX-transfected ovarian cancer stem cells. The results demonstrated that the WWOX gene can be stably expressed in ovarian cancer stem cells and inhibits the proliferation of ovarian cancer stem cells.

Subsequently, flow cytometry was used to investigate the cell cycle of ovarian cancer stem cells. The results demonstrated that following transfection of the WWOX gene, the cell cycle of ovarian cancer stem cells was arrested in the G0/G1 phase, indicating that the WWOX gene significantly inhibits the cell cycle of ovarian cancer stem cells.

The study by Xue *et al* (26) suggests that cancer is a disease of the cell cycle and the occurrence of tumors is closely associated with abnormal cell cycle regulation, where cyclin E-CDK2, cyclin Dl-CDK4 and other cell cycle regulatory proteins are involved. Cyclin E is located on human chromosome 19q12-13 and encodes a 50 kDa protein containing a cyclin box to interact with CDKs. Cyclin-dependent kinase 2 (CDK2) is located on human chromosome 12q13 and encodes a 33 kDa protein of a threonine kinase. Cyclin E and CDK2 are important regulatory factors in the Gl and S phases of the cell cycle (27). Cyclin E binds to CDK2 to form the cyclin E-CDK2 complex, which is a key kinase complex and is important in transition from the G1 to S phase. Expression abnormalities in cyclin E and CDK2 are closely associated with the occurrence and development of breast cancer, stomach cancer, colon cancer and other types of tumor (28,29).

The cyclin D1 gene is located on the long arm of human chromosome 11, area 13 (11q13). In mid-G1 phase, the expressed cyclin Dl protein binds CDK4 to activate and maintain CDK4 activity and promotes cell cycle progression (30). The CDK4 gene is located on the long arm of human chromosome 12, areas 13–14 (12q13-14) and is the regulatory center for the entry of G1 to S phase. When cyclin D1 binds CDK4, Rb kinase is activated to phosphorylate Rb protein, and hyperphosphorylation of Rb protein exerts its transcriptional effect following conformational changes, promoting cells to complete transition of Gl/S phase and to enter the proliferative state (31).

To further investigate the effect and mechanism of the WWOX gene on the cell cycle of ovarian cancer stem cells, the present study used western blot analysis to detect cell cycle-associated proteins in ovarian cancer stem cells. The results demonstrated that following transfection of the WWOX gene, ovarian cancer stem cells expressed significantly lower levels of cyclin E, CDK2, cyclin D1 and CDK4 proteins than the empty plasmid group and the control group. These results indicate that the WWOX gene may downregulate the expression levels of cyclin E-CDK2 and cyclin D1-CDK4 affecting the cell cycle of ovarian cancer stem cells.

Subsequently, flow cytometry was used to detect apoptosis in ovarian cancer stem cells. The results demonstrated that following transfection of the WWOX gene, the rate of apoptosis in ovarian cancer stem cells was significantly increased. These data indicate that the WWOX gene can promote apoptosis of ovarian cancer stem cells.

A previous study demonstrated that Wnt-5α, JNK and caspase-3 are important in the apoptotic process of tumor cells (32). The Wnt signaling pathway induces apoptosis in a variety of tumor stem cells *in vivo*, regulates the proliferation of a variety of tumor stem cells and is important in tumorigenesis and development. Currently, the canonical Wnt signal transduction pathways is being extensively investigated and is considered to be a major factor affecting tumor stem cells. However, studies by McDonald *et al* (33) and Nomachi *et al* (34) suggest that the JNK signal transduction pathway is also closely associated with the occurrence and development of tumor stem cells and the non-canonical Wnt signaling pathway mediated by the JNK pathway also has significant effects on tumor stem cells. The Wnt/JNK signaling pathway is important in multiple processes, including cell proliferation, promotion of apoptosis, mediation of the inflammatory response and gene expression. Caspase-3 is a convergent point of signal delivery from multiple apoptotic stimuli and is considered to be the most important and effective caspase. Caspase 3 is a downstream substrate of the apoptotic Wnt/JNK signaling pathway and its activation is a sign of irreversible apoptosis (35,36).

Therefore, in order to further examine the mechanism underlying the effect of WWOX on the apoptosis of ovarian stem cells, western blot analysis was used to assess apoptosis-associated factors in ovarian cancer stem cells. The results demonstrated that following transfection of the WWOX gene, ovarian cancer stem cells expressed significantly higher levels of Wnt-5α, JNK and caspase-3 mRNA, indicating activation of the Wnt/JNK/caspase-3 signaling pathway. Furthermore, the WWOX gene may promote apoptosis of ovarian cancer stem cells by upregulating the expression levels of Wnt-5α, JNK and caspase-3.

In the present study, the experimental results suggest that the WWOX gene may downregulate the protein expression levels of cyclin E-CDK2 and cyclin D1-CDK4 to affect cell proliferation and the cell cycle of ovarian cancer stem cells. By contrast, the WWOX gene may upregulate the expression levels of Wnt-5α, JNK and caspase-3 to promote apoptosis of ovarian cancer stem cells. Further studies are required to elucidate what the specific target of the WWOX gene is and its role in regulating cell cycle and apoptosis of ovarian cancer stem cells. These findings demonstrated that the WWOX gene may be an important molecular target for the treatment of ovarian cancer in the future

## Figures and Tables

**Table I:** Cell cycle in each group of cells following transfection of the WWOX gene ( $\bar{x}$±standard deviation,%).

Group	G_0_/G_1_ phase	S phase	G_2_/M phase
Recombinant plasmid	65.07±0.60	27.52±0.60	7.61±0.18
Empty plasmid	33.87±0.84	58.03±0.72	8.15±0.54
Blank control	33.69±1.28	57.87±0.71	8.24±0.31

WWOX, WW domain containing oxidoreductase.
